# Electroencephalography Microstate Alterations in Otogenic Vertigo: A Potential Disease Marker

**DOI:** 10.3389/fnagi.2022.914920

**Published:** 2022-06-03

**Authors:** Yi-Ni Li, Wen Lu, Jie Li, Ming-Xian Li, Jia Fang, Tao Xu, Ti-Fei Yuan, Di Qian, Hai-Bo Shi, Shan-Kai Yin

**Affiliations:** ^1^Department of Otorhinolaryngology Head and Neck Surgery, Shanghai Sixth People’s Hospital Affiliated to Shanghai Jiao Tong University, Shanghai, China; ^2^Department of Anesthesiology, Shanghai Sixth People’s Hospital Affiliated to Shanghai Jiao Tong University, Shanghai, China; ^3^Shanghai Key Laboratory of Psychotic Disorders, Shanghai Mental Health Center, Shanghai Jiao Tong University School of Medicine, Shanghai, China; ^4^Department of Otolaryngology, People’s Hospital of Longhua, Shenzhen, China

**Keywords:** vertigo, EEG, neural network, microstate, support vector machine (SVM)

## Abstract

**Objectives:**

A huge population, especially the elderly, suffers from otogenic vertigo. However, the multi-modal vestibular network changes, secondary to periphery vestibular dysfunction, have not been fully elucidated. We aim to identify potential microstate electroencephalography (EEG) signatures for otogenic vertigo in this study.

**Materials and Methods:**

Patients with recurrent otogenic vertigo and age-matched healthy adults were recruited. We performed 256-channel EEG recording of all participants at resting state. Neuropsychological questionnaires and vestibular function tests were taken as a measurement of patients’ symptoms and severity. We clustered microstates into four classes (A, B, C, and D) and identified their dynamic and syntax alterations of them. These features were further fed into a support vector machine (SVM) classifier to identify microstate signatures for vertigo.

**Results:**

We compared 40 patients to 45 healthy adults, finding an increase in the duration of Microstate A, and both the occurrence and time coverage of Microstate D. The coverage and occurrence of Microstate C decreased significantly, and the probabilities of non-random transitions between Microstate A and D, as well as Microstate B and C, also changed. To distinguish the patients, the SVM classifier, which is built based on these features, got a balanced accuracy of 0.79 with a sensitivity of 0.78 and a specificity of 0.8.

**Conclusion:**

There are several temporal dynamic alterations of EEG microstates in patients with otogenic vertigo, especially in Microstate D, reflecting the underlying process of visual-vestibular reorganization and attention redistribution. This neurophysiological signature of microstates could be used to identify patients with vertigo in the future.

## Introduction

Vertigo is one leading symptom in otolaryngology clinics, affecting more than 15–20% of adults yearly (Neuhauser, 2016). Otogenic vertigo caused by periphery vestibular dysfunction accounts for nearly 60% of these cases (Brandt et al., 2013), with a prevalence of around 6.5% and largely increases with age (Hulse et al., 2019). It is associated with increased risks for chronic balance complaints, falls and fractures, lower quality of life, occupational sick leave, and the economic burden on health systems (Mueller et al., 2014; Neuhauser, 2016; Kovacs et al., 2019; Wang et al., 2019). In clinical practices, there are numerous causes and risk factors for otogenic vertigo, accompanying its complicated clinical courses and varied subjective severity (Cousins et al., 2017). Yet, the neural mechanisms underlying it have not been fully elucidated.

The perception of vertigo involves activation of the vestibular, proprioceptive, and visual systems. Such multisensory convergence and sensorimotor integration occur in a multi-modal cortical network called “the vestibular cortex,” which consists of the parietal operculum, temporoparietal, posterior parietal, frontal, and cingulate cortex (zu Eulenburg et al., 2012). Previous studies reported that patients with chronic dizziness show reduced activation in the core vestibular cortex and altered connectivity among the vestibular cortical network based on the functional magnetic resonance imaging (fMRI) findings (Helmchen et al., 2014; Indovina et al., 2015; Van Ombergen et al., 2017). The functional connectivity between the default mode network and the task-positive network seemed also disturbed, which may result from the sustained utilization of processing capacity by diverging sensory information (Klingner et al., 2014).

When compared to neuroimaging approaches, electroencephalography (EEG) provides a cheap and easy measurement of neural activities with high clinical feasibility and better temporal resolution. The EEG metrics have been taken as both biomarkers for diagnosis and predictors for therapeutic response in several diseases (Gaubert et al., 2019; Wu et al., 2020). Indeed, it is found that otogenic vertigo is associated with alpha-, beta-, and gamma-band activity changes across the vestibular cortex, and the increased alpha-gamma nesting in the left frontal eye field is positively correlated with chronic symptom intensity (Alsalman et al., 2016; Gale et al., 2016; Cha et al., 2021). These studies report preliminary oscillation traits of otogenic vertigo, but their scattered distribution makes it hard to understand the landscape of neural signatures.

Microstates are topography patterns of scalp potential fields, which remain quasi-stable for 60–120 ms, and indicate changes in global network activity (Michel and Koenig, 2018). Considered as “atoms of thought,” only a few dominant microstate topographies were reported to characterize the ongoing broad-band EEG (Wackermann et al., 1993; Lehmann et al., 1998; Michel and Koenig, 2018). Taken as a highly reproducible feature (Khanna et al., 2014; Zhang et al., 2021), EEG microstate has been proposed as a potential endophenotype or biological marker for neurophysiological health and diseases including schizophrenia, dementia, and hallucination (Kindler et al., 2011; Schumacher et al., 2019; da Cruz et al., 2020). A recent study investigated the microstate architecture of healthy volunteers during passive whole-body movement with weak to moderate acceleration intensities and found that there was no significant difference between all conditions (Ertl et al., 2020).

In this study, we aim to identify potential microstate EEG signatures for vertigo. We utilized 256-channel EEG for a 7-min resting-state collection with eyes closed, which provide us with millimeter accuracy in topography signals. Then, we identified four classes of microstates, labeled A, B, C, and D, which are commonly used to describe large-scale brain networks, explaining 65–84% of the global variance (Lehmann et al., 2009; Khanna et al., 2015; Michel and Koenig, 2018). We further establish a machine learning classifier to distinguish the patients with vertigo from healthy adults based on significant EEG features selected by group comparisons. The results showed that the temporal dynamics of microstates, especially Class D, could be candidate neural signatures for vertigo. The findings shall provide insights into the neurobiological mechanisms of vertigo and a potential prediction feature to aid clinical diagnosis.

## Materials and Methods

### Participants

Patients with vertigo and balance complaints were recruited from the Otolaryngology-Head and Neck Surgery Department of the Sixth People’s Hospital Affiliated with Shanghai Jiao Tong University. The inclusion criteria were: (1) age above 18 years old; (2) suffering recurrent vertigo for more than 1 month, and diagnosed as “otogenic vertigo” by experienced professional doctors; (3) could complete the questionnaire and vestibular function tests under the help of doctors and researchers; (4) no recent or long-term history of psychoactive substance use; (5) not co-morbid with severe systemic diseases, psychiatric disorders nor neurological disorders (e.g., brain tumors, epilepsy).

A total of 40 patients were recruited (12 males and 28 females), including 19 Meniere’s Disease, eight residual dizziness after Benign Paroxysmal Positional Vertigo, and 13 other otogenic vertigo diseases. In addition, 45 age-matched healthy volunteers with no history of vertigo attacks were involved in the healthy control (HC) group (14 males and 31 females). All participants were informed of the purpose, potential consequences, benefits, and their right to quit the experiments at any time. Written informed consent was obtained before the study. This study was approved by the Institutional Ethics Review Board of the Sixth People’s Hospital Affiliated with Shanghai Jiao Tong University.

### Questionnaire and Vestibular Testing

The 25-item Dizziness handicap inventory (DHI) was used to quantify the subjective intensity of vestibular symptoms and the self-perceived handicapping effects (Jacobson and Newman, 1990; Ding et al., 2013). As a reliable self-report indicator, DHI is commonly used clinically and consists of three dimensions representing emotional, physical, and functional aspects of handicaps (Perez et al., 2001). DHI score ranges from 0 to 100, with 36 in the emotional aspect, 28 in the physical aspect, and 36 in the functional aspect (Mutlu and Serbetcioglu, 2013). To categorize the intensity, we take total scores ranging from 0 to 30 as a mild handicap, 31–60 as a moderate handicap, and 61–100 as a severe handicap.

The 7-item Generalized Anxiety Disorder Scale (GAD-7) (Spitzer et al., 2006) was used to measure the severity of anxiety, while the 9-item Patient Health Questionnaire subscale (PHQ-9) (Spitzer et al., 1999) was used to measure the severity of depression.

Computerized dynamic posturography (CDP) was used to evaluate the balance performance of all the participants (Equitest, NeuroCom International, Inc., Clackamas, OR, United States) (Palm et al., 2014). The sensory organization test (SOT) was conducted to evaluate the patient’s ability to integrate the visual, vestibular, and somatosensory functions to maintain balance in six increasingly challenging conditions (condition 1–6), which is measured by equilibrium score 1–6 (EC 1–6). EC 1–3 measured patients’ performance on a stable surface with normal or disrupted visual input, while EC 4–6 measured that on an unstable surface. An equilibrium composite score will be given as an overall estimate for postural stability and balance control. Each score approaching 0% represents instability or poorer functions and is considered abnormal when it’s lower than the age-specific normal data.

In addition, the video Head Impulse Test (vHIT) test was conducted to measure the periphery vestibular function of semicircular canals (Otometrics A/S, Taastrup, Denmark).

### Electroencephalography Recording and Data Processing

Patients were instructed to sit comfortably in a sound-shield room, remaining relaxed and awake during the whole recording. The high-density EEG was acquired with 256-channel EEG Geodesic Net Amps (Electrical Geodesics, Eugene, OR, United States) at a sampling rate of 1,000 Hz. The recording lasted for 14 min, including 7 min with eyes closed and 7 min with eyes open. Only eyes-closed EEG data were analyzed.

Offline data were downsampled to 500 Hz and preprocessed in Matlab (Mathworks) using the EEGlab toolbox (Delorme and Makeig, 2004). The data were filtered with a bandpass of 0.5–50 Hz, topographically interpolated bad channels, segmented into 2-s segments, and re-referenced to the grand average. The artifacts including eye movement, heartbeat, and muscle artifacts were removed by independent component analysis (ICA).

### Microstate Analysis

The microstate analysis was performed in Matlab (Mathworks) using the EEGlab plug-in Microstate Analysis (Version 0.3, Thomas König, University of Bern, Switzerland). Then, the cleaned EEG data were band-pass filtered between 2 and 20 Hz. The maxima of the Global Field Power (GFP) were determined, and the polarity was ignored. To get the best signal-to-noise ratio, only EEG topographies at the GFP peaks were extracted and clustered into four classes within subjects using the “atomize and agglomerate hierarchical clustering” (AAHC) algorithm (Koenig et al., 2002; von Wegner et al., 2018). Also, we further clustered the individual template maps of each subject through a second AAHC cluster analysis to get the group template. Then, we computed the spatial correlation between the group template and the subject template, labeling each map with the best-corresponded group template. At last, we evaluated the spatial correlation between EEG and the templated maps to obtain a measure of how well our templates explained the EEG topography at every time point (Murray et al., 2008; Britz et al., 2010).

The following microstate parameters were computed: (1) mean duration, (2) time coverage, (3) frequency of occurrence, and (4) transition probability to a specific microstate class. Mean duration (in ms) is the average time that a given microstate was uninterruptedly present. Time coverage (in %) is the percentage of the total analysis time spent in each microstate. Occurrence is the mean number of times a given microstate is occurring per second.

### Machine Learning

Considering the wide use of the support vector machine (SVM) for classification in multiple brain disordered research (Gao et al., 2018; Betrouni et al., 2019; Pisner and Schnyer, 2020; Kinreich et al., 2021), we adopted SVM and build a classification model to distinguish the patients with vertigo and healthy controls. The microstate features were mainly selected by group comparisons between patients with vertigo and healthy adults. The correlations between these features and clinical parameters were also considered. Also, the features were finally determined by a backward strategy. The quantitative data were normalized in advance to reduce the effect of the range of values. To get more explainability, we chose a linear kernel to build our model. The penalty parameter C was optimized by a 5-fold grid search process. Considering our limited sample size, we chose a leave-one-out nested cross-validation framework to train our model and estimate its performance. The accuracy, sensitivity, specificity, and balanced accuracy were adopted to measure its performance, but only the accuracy was used in the optimization of parameters. The balanced accuracy was calculated as the average of sensitivity and specificity.

### Statistical Analysis

The group differences in microstate parameters were investigated using the unpaired *t*-test or Mann-Whitney test separately for each microstate parameter, depending on the distribution of data. A *p* level of less than 0.05 (two-sided) was considered to be statistically significant. In syntax analysis of between-group difference, a repeated measured (rm)-ANOVA with *post hoc* group comparisons was applied. Also, Bonferroni’s correction was used for correcting multiple comparisons. To investigate the correlation between microstate parameters and clinical features, we performed Pearson or Spearman correlation based on whether the data distribution is normal. The analysis was also conducted separately for each parameter within the patients with vertigo group. Statistical tests were performed with Python (version 3.8.8) and GraphPad Prism Software (San Diego, CA, United States).

## Results

The characterizations of patients with otogenic vertigo, including balance performance and questionnaire scores, are presented in Table 1. The healthy controls report no vertigo attacks by now and their DHI scores are all 0. No significant differences were found between patients with vertigo and the healthy control group in age (*t* = 0.046, *df* = 83, *p* = 0.964) and gender (*x*^2^ = 0.012, *df* = 1, *p* = 0.912).

**TABLE 1:** Demographic and clinical characteristics of patients with otogenic vertigo.

	Mean ± SD/Median (IQR)
Age	51.5 ± 12.9
Gender (M/F)	12/28
Disease duration (m)	37.6 ± 61.2
DHI	45.1 ± 19.3
DHI emotional	13.9 ± 7.6
DHI physical	13.3 ± 6.5
DHI functional	18.0 ± 8.7
PHQ-9	6.5 ± 9.0
GAD-7	4.5 ± 5.0
SOT equilibrium scores	71.5 ± 8.2
EC1	93.0 (3.5)
EC2	91.5 (4.5)
EC3	89.0 (5.5)
EC4	75.0 (13.5)
EC5	59.0 (19.5)
EC6	51.5 (23.0)

### Microstate Signatures in Patients With Vertigo

**FIGURE 1:**
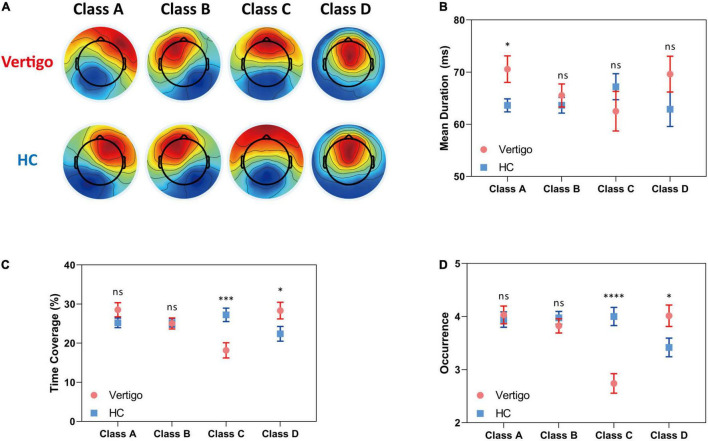
Results of the microstate analysis for patients with vertigo and healthy controls. Data of patients with vertigo are displayed in red and controls in blue. **(A)** Global architecture of the four microstates **(A–D)** for both groups. Group average parameters **(B)** mean duration, **(C)** time coverage, and **(D)** occurrence frequency of each class for patients versus healthy controls. Error bars represent SEM. ns, not significant, *p < 0.05, ^***^p < 0.001, ^****^p < 0.0001 by unpaired *t*-test.

Similar to pioneering works: we clustered the microstates into four classes: A, B, C, and D. These classes, respectively, explained 76.64 and 78.01% of the global variance in patients with vertigo and healthy controls across all participants. The grand mean microstate topographies clustered from two groups were shown in Figure 1A. For each participant, the microstate parameters were computed, including mean duration, time coverage, and occurrence frequency. In addition, the syntax analysis was also performed to identify the potential non-random transfer sequence.

**FIGURE 2:**
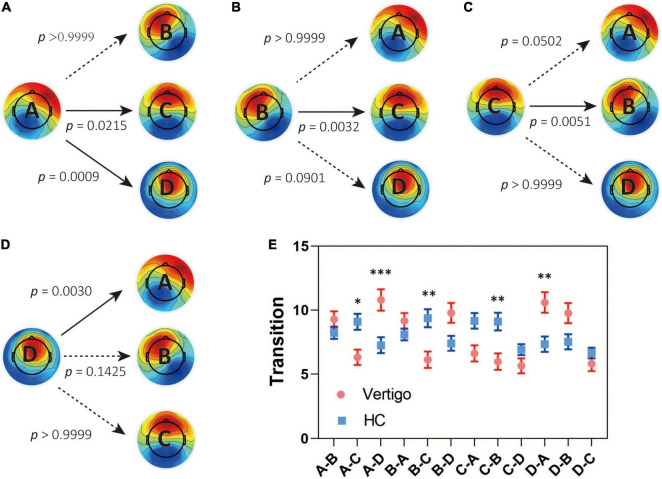
Results for the syntax analysis between patients with vertigo and healthy controls. Transitions from **(A)** Class A, **(B)** Class B, **(C)** Class C, **(D)** Class D to the other three classes. **(E)** The transitions in both groups and the between-group difference in syntax analysis. Data of patients with vertigo are displayed in red and controls in blue. Solid arrows indicate significant differences. *p < 0.05, ^**^p < 0.01, ^***^p < 0.001 by *post hoc* pairwise comparisons with Bonferroni’s correction for 12 comparisons.

In seeking the microstate alterations in patients with vertigo, we performed a group comparison for each parameter. Group average templates are illustrated in Figures 1B–D and the *t*-tests for all three parameters between two groups are given in Table 2. The duration of Microstate A increased slightly in patients with vertigo, while the time coverage and occurrence frequency appeared to be constant. In addition, the time coverage and occurrence significantly decreased for Microstate C, while increased for Microstate D (all, *p* < 0.05). There was no significant difference in Microstate B in terms of duration, coverage, and occurrence. As for the transition probability, we found a higher probability for transition into Microstate D and a lower probability for transition into Microstate C, both of which initiate mainly from Microstate A and Microstate B. The transition between Microstate C and D remained relatively constant. In addition, the transition from Microstate C into Microstate A and Microstate B decreased in patients with vertigo. The differences were shown in Figure 2.

**TABLE 2:** Patients with vertigo versus healthy controls for each microstate class.

Parameter	Micostate	Vertigo	HC	*p*-Value
Mean duration (ms)	Class A	70.57 ± 16.02	63.62 ± 8.39	**0.0129**
	Class B	65.52 ± 14.03	63.64 ± 10.10	0.4764
	Class C	62.51 ± 24.02	67.20 ± 16.75	0.2954
	Class D	69.62 ± 21.68	62.88 ± 22.19	0.1614
Time coverage (%)	Class A	28.53 ± 11.46	25.19 ± 8.21	0.1229
	Class B	25.02 ± 8.95	25.22 ± 7.37	0.9094
	Class C	18.14 ± 12.32	27.22 ± 11.60	**0.0007**
	Class D	28.31 ± 13.42	22.37 ± 12.70	**0.0389**
Occurrence	Class A	4.03 ± 1.06	3.94 ± 0.97	0.6905
	Class B	3.82 ± 0.85	3.97 ± 0.81	0.4029
	Class C	2.74 ± 1.17	4.00 ± 1.15	**<0.0001**
	Class D	4.01 ± 1.28	3.42 ± 1.18	**0.0275**

*p-Values refer to unpaired t-test p-value for each parameter separately between patients with vertigo and healthy controls. Statistically significant differences are in bold.*

### Microstate Signatures Correlate With Subjective Vertigo Intensity and Vestibular Function

**FIGURE 3:**
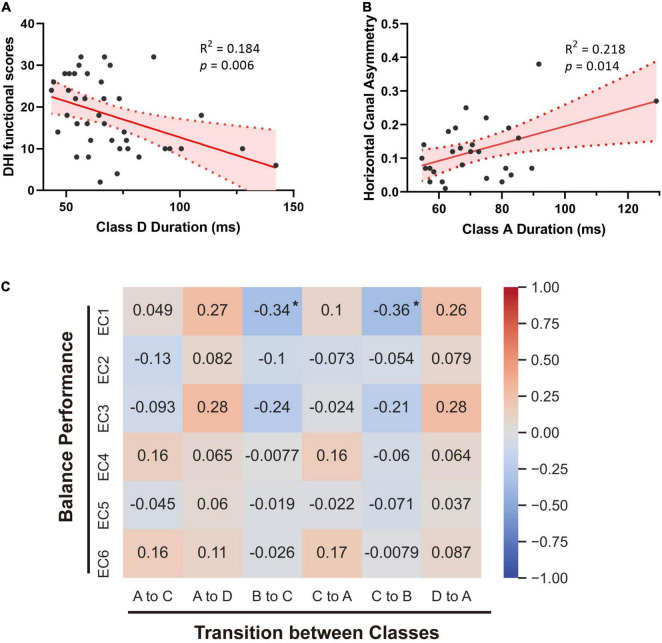
Correlations between microstates and clinical parameters. **(A)** Linear regression analysis of Class D duration and the functional handicaps measured by DHI. **(B)** Linear correlation between Class A duration and the asymmetry of horizontal semicircular canal gains. **(C)** Spearman’s correlations between microstates’ transitions and balance performance of patients with vertigo. Spearman R values are marked. *p < 0.05.

To provide further insight into the underlying clinical meaning of such alterations, we investigated the correlations between these parameters and clinical characteristics within patients with vertigo, including DHI scores, balance performance, disease duration, GAD7 scores, and PHQ9 scores. No significant correlation was found between the microstate alterations and emotional questionnaire scores. The subjective severity of vertigo measured by DHI was positively correlated with Microstate B Occurrence in patients with vertigo (*p* = 0.036, *R* = 0.332). The functional aspect of DHI was found negatively correlated with Microstate D duration (*p* = 0.006, *R* = −0.428; Figure 3A). The transition from Class B into Class C and the reverse transition were both found to negatively correlate with EC1 (*p* = 0.035, *R* = −0.338; *p* = 0.026, *R* = −0.357; Figure 3C). In addition, the duration of Class A is positively correlated with the asymmetry of horizontal semicircular canal gains (*p* = 0.014, *R* = 0.218; Figure 3B). Notably, the most obvious alterations in the occurrence and time coverage of Class C and D showed no significant correlations with DHI or balance performance.

### Classification Performance in Support Vector Machine Classifier Based on Microstate Signatures

**FIGURE 4:**
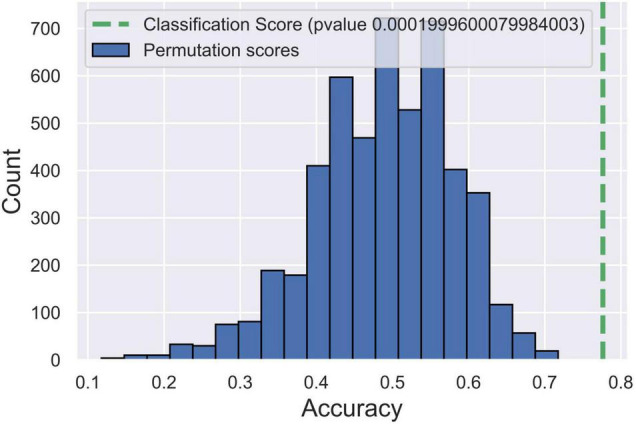
*Post hoc* test for support vector machine (SVM) model *via* permutation testing. The green dotted line indicates the accuracy of our SVM model. Blue histograms indicate the accuracy distribution of SVM classifications in 5,000 randomly permutated datasets.

To identify the microstate markers for patients with vertigo, we fed the signatures above to a linear SVM classifier. With feature reduction and model optimized by a backward strategy, we take 10 features at last, including age, gender, Class A duration, the occurrence of Class C and D, time coverage of Class D, the transition between Class A and D, the transition from B to C, and that from C to A. The confusion matrix was shown in Table 3, the accuracy was 0.79, the sensitivity was 0.78, and the specificity was 0.8, indicating its effectiveness in distinguishing patients with vertigo. Then, we tested the statistical significance of the sensitivity and specificity *via* permutation testing (*P* < 0.001; Figure 4). We also analyzed further the SVM weights to identify the features that made the greatest contribution to the classification. The transition from D to A, and the occurrence of Classes C and D were the three most important features, followed by the duration of Class A and the time coverage of Class D.

**TABLE 3:** Confusion matrix of support vector machine classification.

	HC	Vertigo
HC (predicted)	36	9
Vertigo (predicted)	9	31
Total	45	40

## Discussion

The present study reported the topography and dynamics of microstates in patients with otogenic vertigo, which established a global landscape of neural network transition at high temporal resolution. Such characteristic alterations of resting-state EEG microstates could indicate neural network changes underlying vertigo, and potentially aid in clinical diagnosis and prognosis.

Recently, Ertl et al. (2020) reported that the EEG microstate of 29 healthy volunteers seemed invariant during passive body translations with weak whole-body accelerations. With microstates clustered into the same four classes as us, their duration of Class A, time coverage of Classes C and D, and the occurrence of Classes C and D seemed to be between those of our patients with vertigo and healthy controls. It indicates that microstates of healthy people may show the same tendency during vestibular stimulus as patients with vertigo in a resting state. As for the obvious increase in the duration of Class B, it may be caused by the eye-open state in Ertl’s experiments, which could lead to increased activation of visual systems. This consistency between these studies further suggests the potential of basic microstate parameters to distinguish vertigo.

In this study, we found that the duration of Class A is longer in patients with vertigo and there is a positive correlation between such an increase and periphery vestibular function loss (Figures 1B, 3B). Microstate A was reported to be correlated with negatively BOLD activation in bilateral superior and middle temporal gyrus (Michel and Koenig, 2018), which were involved in the auditory network and the multi-modal vestibular network. It is consistent with the reversible decrease reported in resting-state activity during vestibular neuritis parallel to the disease progression (zu Eulenburg et al., 2012; Helmchen et al., 2014). Furthermore, we also found that the duration of Microstate D tends to be higher in patients with vertigo than in healthy controls, despite its statistical insignificance. However, unlike that of Microstate A, its negative correlation with patients’ functional handicaps indicates that such an increase could be a central compensatory mechanism (Figures 1B, 3A). The pronounced individual variability in these increases may reflect the heterogeneity in the degree and strategies of the compensation.

As for the syntax analysis, the non-random transition probabilities may suggest an encoded sequential activation of neural assemblies (Khanna et al., 2015). There is an increased transition between Microstate A and D and a decreased transition between Microstates B and C in patients with vertigo (Figure 3). According to previous studies, Microstates B, C, and D are separately associated with the visual system, the task-negative network, and the dorsal attention network (Britz et al., 2010; Seitzman et al., 2017). These altered transition probabilities in patients with vertigo may reflect the compensatory plasticity by combing visual and vestibular function with cognitive function, raising a requirement for more cognitive resources in information integration. This is also supported by the decreased coverage and occurrence of Microstate C (Figures 1C,D), which implies the inhibition of task-negative network and more cognitive need in long-term balance challenges. The increase in compensational tendency in Microstate D, as mentioned above, is also consistent with this. In addition, Microstate C is associated with the activation of the anterior cingulate and insula, which are referred to as the cingulo-opercular system (Seeley et al., 2007; Coste and Kleinschmidt, 2016) and are regarded crucial in the vestibular cortex (zu Eulenburg et al., 2012). Thus, the decrease of transition between Microstates C and B could also imply that the patients with vertigo tend to re-weigh the sensory input and rely less on vestibular information as a compensatory strategy. Notably, patients with vestibular disorders were reported to experience cognitive difficulties in attention, space and time perception, visuospatial processing, and executive function regardless of gender or emotional state (Pineault et al., 2020; Xie et al., 2022). Such cognitive dysfunction was reported to be correlated with vertigo severity and symptom durations (Rizk et al., 2020). The cognitive-motor interference has been proposed as a potential explanation for such cognitive impairments, which also emphasizes the decline in cognitive reserve caused by the increased amount of mental capacity required in balance control (Danneels et al., 2020). As for the elderly, the age-related vestibular impairment may lead to poorer cognitive abilities, or even contribute to a “spatial subtype” of Alzheimer’s disease (Agrawal et al., 2020).

Considering the pronounced individual variability in both clinical and EEG features, it is necessary to realize that vertigo could be too heterogeneous to descript in simple parameters. Thus, there is a clinical need for a new biomarker combination. Different patterns of microstate transitions have been proposed as an endophenotype or a biomarker for neuropsychiatric diseases in many studies (Khanna et al., 2015; Michel and Koenig, 2018; Schumacher et al., 2019; da Cruz et al., 2020). So, we propose that resting-state EEG microstates could also offer potential biomarkers for vertigo. Machine learning methods have been widely applied in diagnosis or prediction tasks, holding promise for building innovative models based on various features, so we established an SVM classifier. Lee et al. (2018) reported an SVM model based on 11 fMRI functional connectivity parameters in previous studies, with a specificity of 0.769 and a sensitivity of 0.784. The slightly higher efficacy of our model indicates that the microstates are promising in distinguishing patients with vertigo. Considering our limited sample size and our lack of balance performance or psychoneurological questionnaires in healthy controls, it may be more powerful with all aspects included in the future. In addition, with a linear kernel, we could identify the most important microstate signatures by the SVM weight vector. As mentioned above, the transition from D to A, and the occurrence of Classes C and D were the three most important signatures, highlighting the significance of visual-vestibular reorganization accompanied by attention redistribution.

Although we identified these microstate signatures for otogenic vertigo, it is worth noting that we still should be cautious to interpret the microstate results, considering our lack of a full understanding of the significance and function details of both EEG microstates and fMRI resting-state networks. When it comes to vertigo diseases, the lack of previous studies makes it even more difficult to interpret. More studies of EEG changes in different vertigo types might reveal valuable insights. Besides, the microstate signatures identified in our study should be explored in longitudinal studies to further elucidate their significance as predictors. Furthermore, whether and how the alterations of microstates will influence the responses of patients with vertigo to medical treatment should also be investigated in the future.

In sum, the study firstly reported the temporal dynamic changes of EEG microstates in patients with otogenic vertigo. There were several significant alterations in the dynamics and syntax of resting-state EEG microstates among these patients, which correlated to clinical balance performance. The results provide potential neurophysiological signatures for future otogenic vertigo diagnosis.

## Data Availability Statement

The raw data supporting the conclusions of this article will be made available by the authors, without undue reservation.

## Ethics Statement

The studies involving human participants were reviewed and approved by the Institutional Ethics Review Board of the Sixth People’s Hospital Affiliated to Shanghai Jiao Tong University. The patients/participants provided their written informed consent to participate in this study.

## Author Contributions

Y-NL, H-BS, T-FY, DQ, and S-KY conceived and designed the study. Y-NL, WL, JL, and TX collected the EEG data. Y-NL, WL, JL, M-XL, and JF collected the clinical data. Y-NL, WL, and H-BS analyzed the data and wrote the manuscript together. DQ and T-FY contributed to the manuscript revisions. All authors contributed to the article and approved the submitted version.

## Conflict of Interest

The authors declare that the research was conducted in the absence of any commercial or financial relationships that could be construed as a potential conflict of interest.

## Publisher’s Note

All claims expressed in this article are solely those of the authors and do not necessarily represent those of their affiliated organizations, or those of the publisher, the editors and the reviewers. Any product that may be evaluated in this article, or claim that may be made by its manufacturer, is not guaranteed or endorsed by the publisher.
